# Supplementary material for: Non-catalytic role of phosphoinositide 3-kinase in mesenchymal cell migration through non-canonical induction of p85β/AP2-mediated endocytosis
Source: Nat Commun. 2024 Mar 23;15:2612. doi: 10.1038/s41467-024-46855-y (PMC10960865; doi:10.1038/s41467-024-46855-y)
Supplement: Supplementary file 1 — Supplementary Information [file 41467_2024_46855_MOESM1_ESM.pdf]

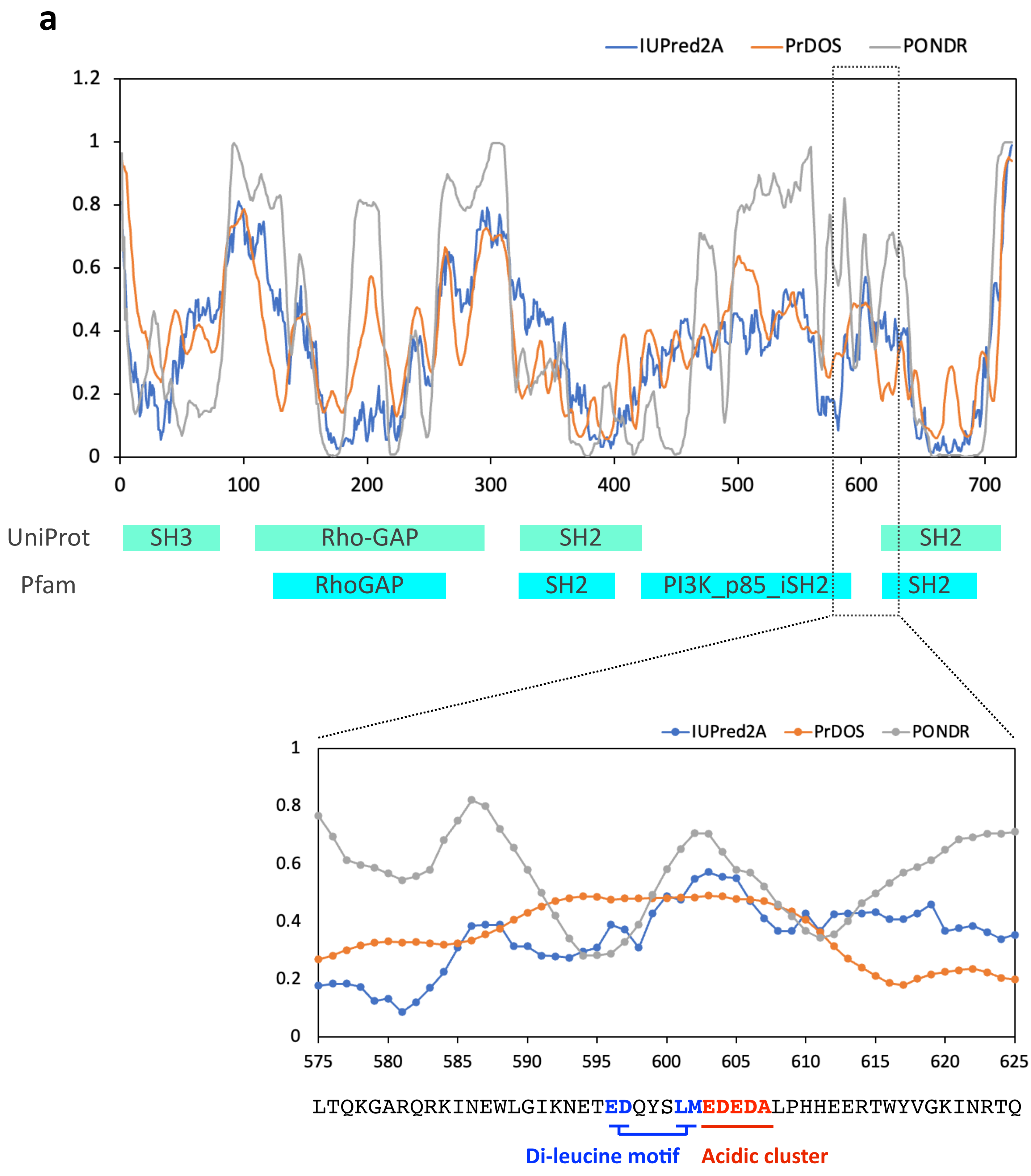

Supplementary Figure 1

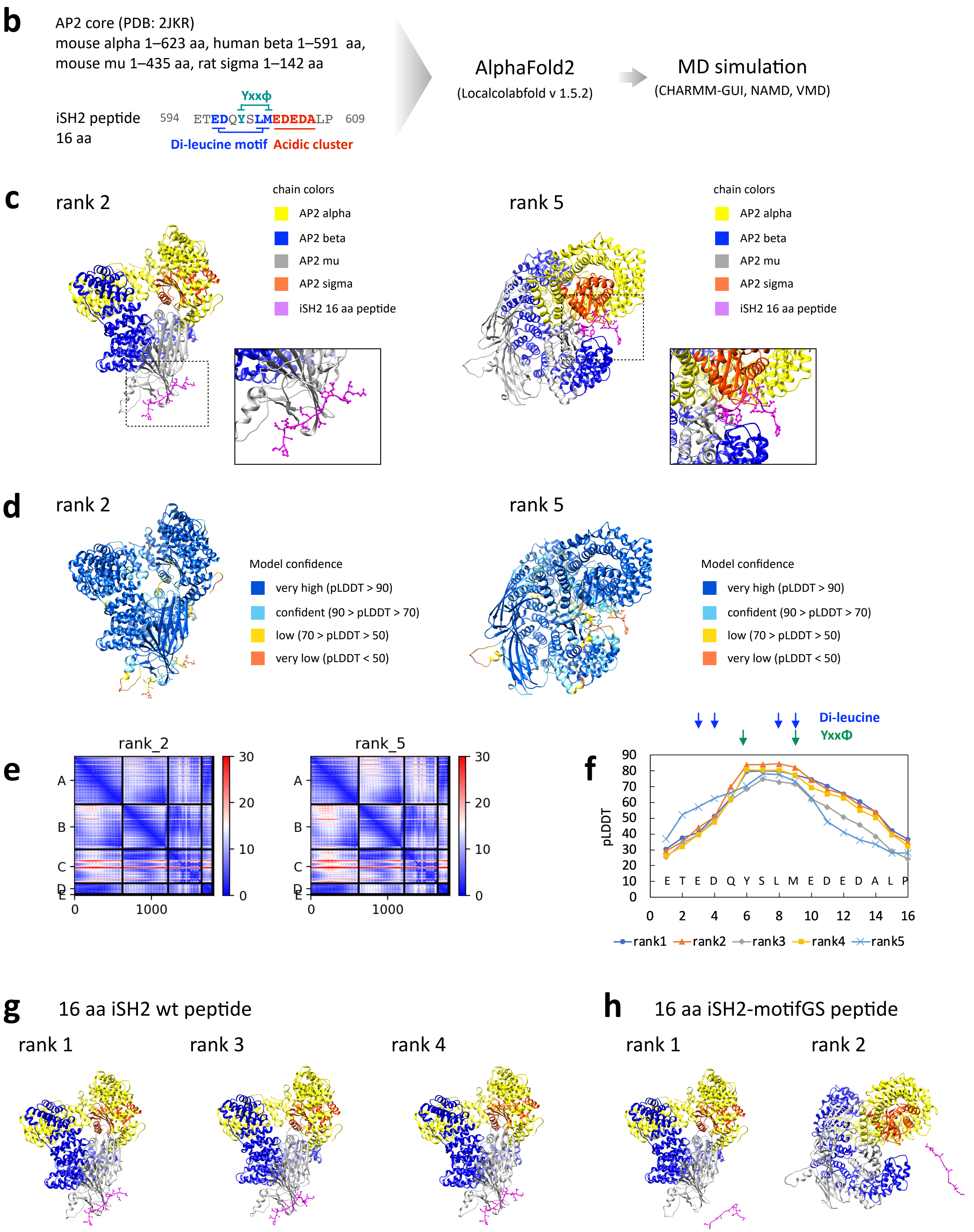

Supplementary Figure 1

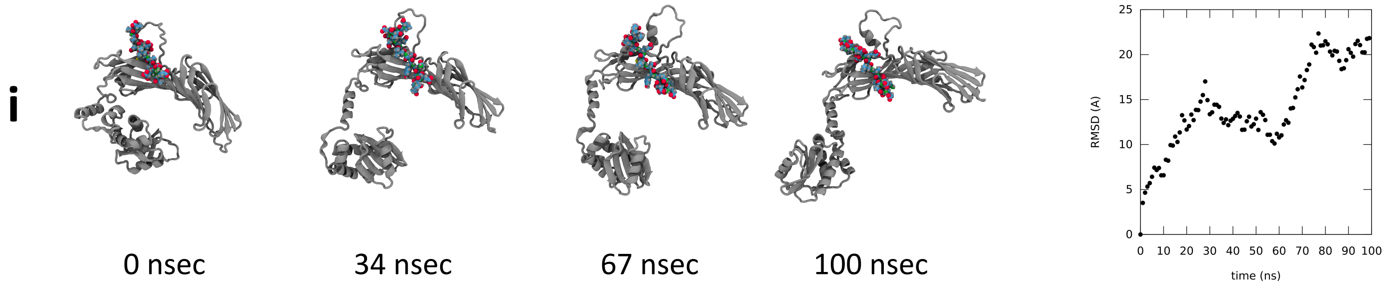

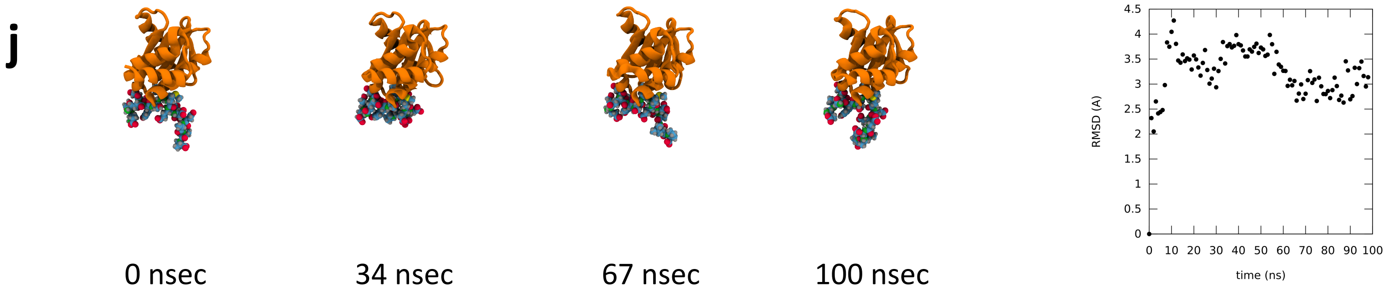

Supplementary Figure 1: Prediction of intrinsically disordered regions. (a) Intrinsically disordered region of mouse p85β (PIK3R2) was analyzed by three algorithms, IUPred2A, PrDOS, and PONDR. (b) Schematic of AF2 computation and MD simulation. (c) Structure of rank 2 (μ-YxxΦ binding) and rank 5 (σ-Di-leuciine binding) AF2 predictions. Insets are zoomed up views of iSH2 peptide binding sites. (d) AF2 predicted structures colored by model confidence (pLDDT). (e) Predicted Aligned Error (PAE) plots of rank 2 and 5 structures. (f) pLDDT plot of 16 aa iSH2 peptide in each AF2 predictions. (g) The other AF2 predicted structures (rank 1, 3, 4). (h) Two representative AF2 structures (rank 1 and rank 2) predicted from AP2 core and motifGS 16 aa iSH2 peptide (ETSAGGSAGGSAGGLP). Structures show that MotifGS iSH2 peptide was assigned in distant positions. (I, j) All-atom MD simulation results and the time course of Root-mean-square deviation (RMSD). In MD simulations, AP2 μ subunit and iSH2 peptide were taken from rank 2 structure in (i) and AP2 σ subunit and iSH2 peptide were taken from rank 5 in (j), respectively. Gray: μ subunit, Orange: σ subunit, ball-stick representation: 16 aa iSH2 peptide. RMSD was calculated for all the protein-peptide atoms simulated in the analysis compared to the initial configuration.

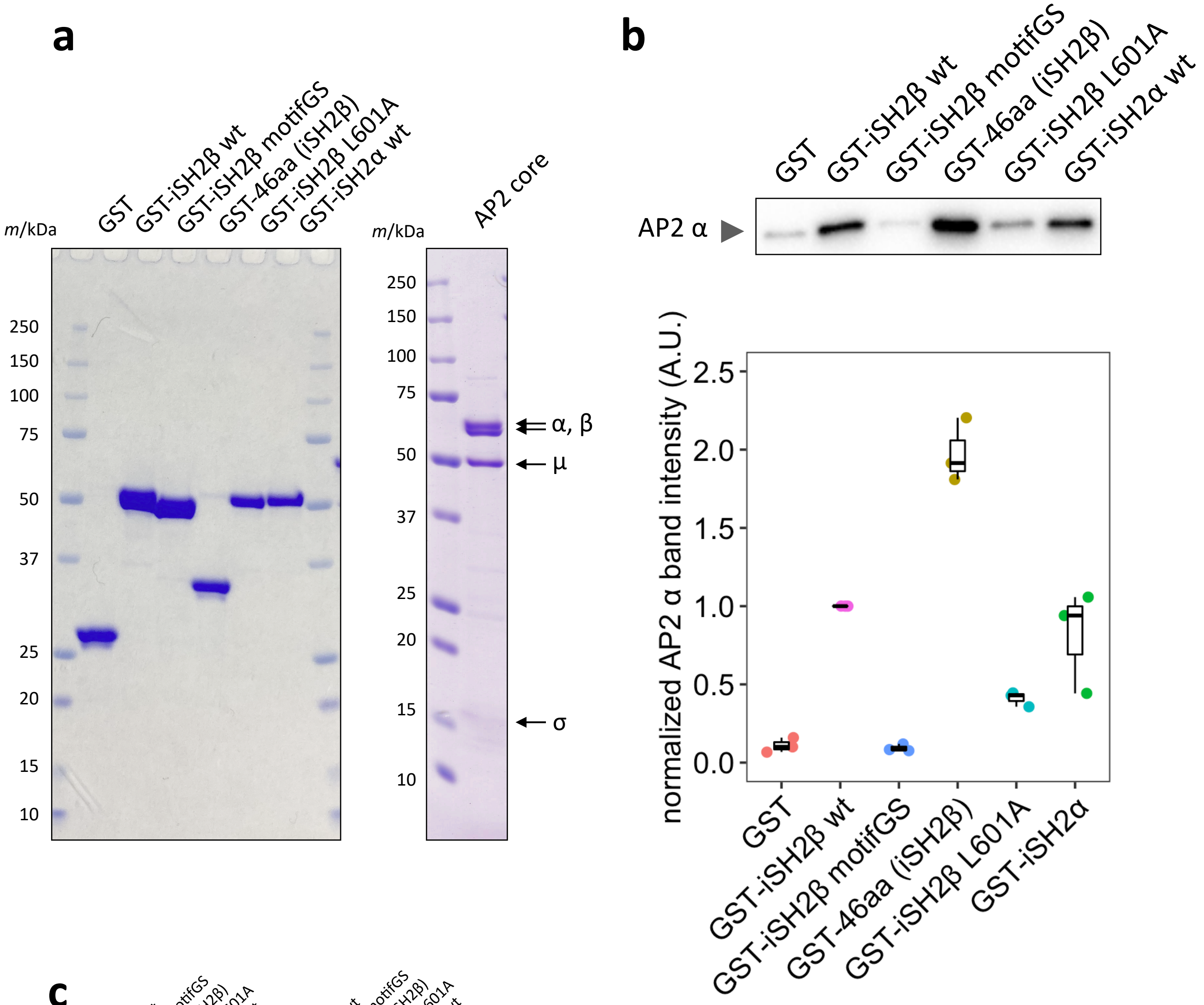

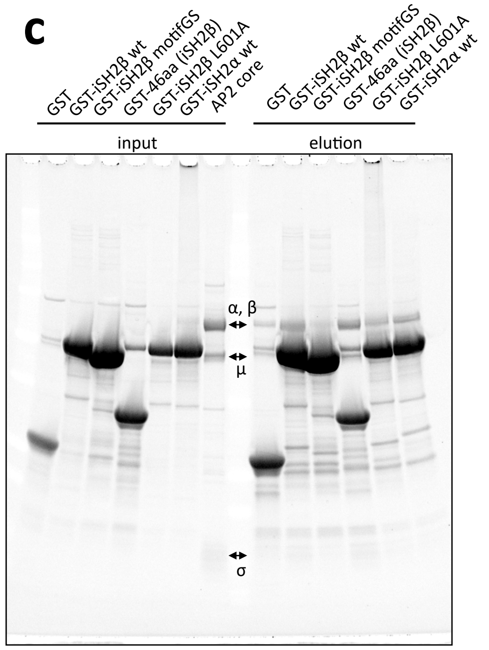

Supplementary Figure 2: iSH2-AP2 pulldown. (a) SDS-PAGE results of GST-iSH2 variants and AP2 core used in pulldown assay (GST: 30.3 kDa, GST-iSH2β wt: 53.5 kDa, GST-iSH2β motifGS: 52.9 kDa, GST-46aa (iSH2β): 35.3 kDa, GST-iSH2β L601A: 53.5 kDa, GST-iSH2α wt; 53.7 kDa, AP2 α: 70.3 kDa, AP2 β: 67.9 kDa, AP2 μ: 49.7 kDa, AP2 σ: 17.0 kDa). (b) Top: western blot of AP2 α (different data set from Fig. 1f). Bottom: quantification of pulled down AP2 α. Band intensity was normalized by the band in GST-iSH2β wt lane in each experiment. Box whisker plots represent median, 1st, 3rd quartiles and 1.5×inter-quartile range. (c) SYPRO Ruby staining of pulldown SDS-PAGE. input: purified proteins used in the assay. elution: 20 mM glutathione elution fractions.

Supplementary Figure 2

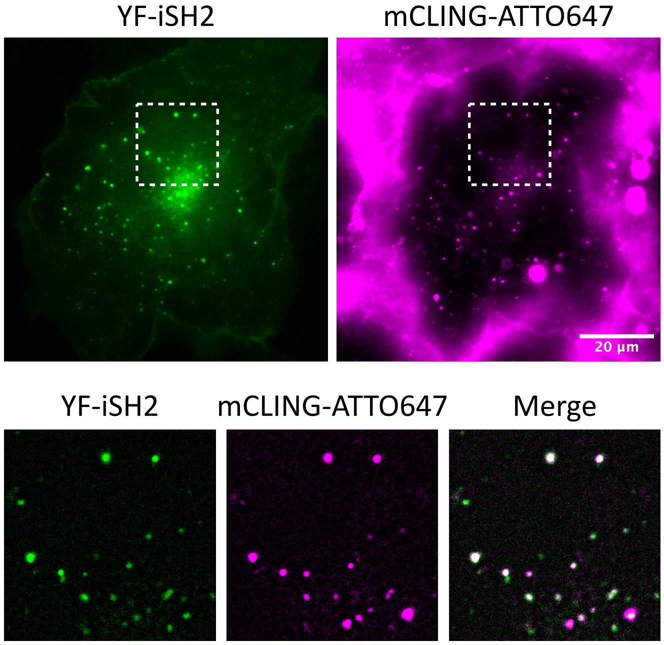

Supplementary Figure 3: iSH2-vesicles colocalize with mCLING dye. Epi-fluorescence microscopy images of iSH2-vesicles colocalized with extracellularly added mCLING-ATTO647. Cos7 cells were transiently transfected with Lyn-ECFP-FRB, EYFP-FKBP-iSH2, mCherry-PH(Akt). After mCLING addition, iSH2 translocation and vesicle formation was induced by 100 nM rapamycin. 30 min after rapamycin addition, the samples were chilled, washed, and fixed with 4% paraformaldehyde. Top: raw image of a transfected cell. Bottom: enlarged images of dashed line area of top images. To reduce background noise, median filtered values were subtracted from the raw images.

Supplementary Figure 3

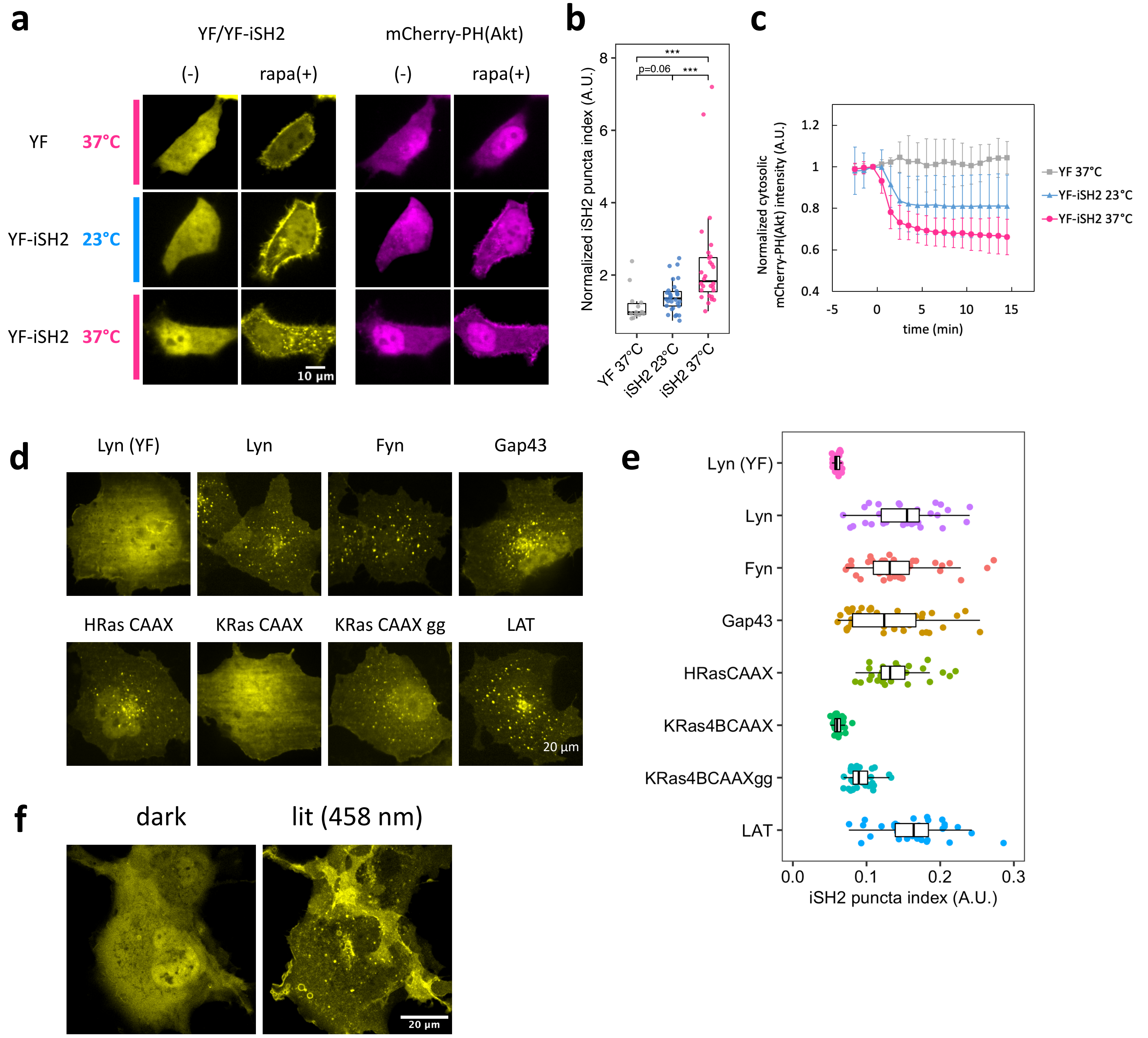

Supplementary Figure 4: iSH2-mediated endocytosis is temperature dependent but independent of membrane targeting or dimerization scheme. (a) Confocal images of endocytic vesicle production and PH(Akt) translocation. HeLa cells were transiently transfected with Lyn-ECFP-FRB, mCherry-PH(Akt), and EYFP-FKBP or EYFP-FKBP-iSH2. (-) before rapamycin addition, rapa(+) 20 min after adding 100 nM rapamycin. (b) Quantified iSH2-mediated endocytosis indices. The values were normalized by time=0. Box whisker plots represent median, 1st, 3rd quartiles and 1.5×inter-quartile range. P-value ***: < 0.001. Steel-Dwass test. (c) Time course of PH(Akt) translocation. Cytosolic intensity of mCherry-PH(Akt) was quantified and normalized by time=0. Error bars represent standard deviation. YF 37°C, n=15 cells. YF-iSH2 23°C, n=30 cells. YF-iSH2 37°C, n=28 cells. (d, e) Confocal images of iSH2-vesicles produced with different plasma membrane anchors and the quantified iSH2 puncta index. Cos7 cells were transiently transfected with EYFP-FKBP-iSH2, mCherry-PH(Akt), and ECFP-FRB fused with different types of plasma membrane anchors. 15 min after adding 100 nM rapamycin, cells were chilled, washed, and fixed with 4% paraformaldehyde and 0.15% glutaraldehyde. Box whisker plots represent median, 1st, 3rd quartiles and 1.5×inter-quartile range. (f) Confocal images of iSH2-vesicles induced by iLID/SspB system. Cos7 cells were transiently transfected with Lyn-iLID and EYFP-SspB-iSH2. dark: before light stimulation. lit (458 nm): 15 min after 458 nm light illumination. EYFP-SspB-iSH2 shows punctate structure in the cytosol.

Supplementary Figure 4

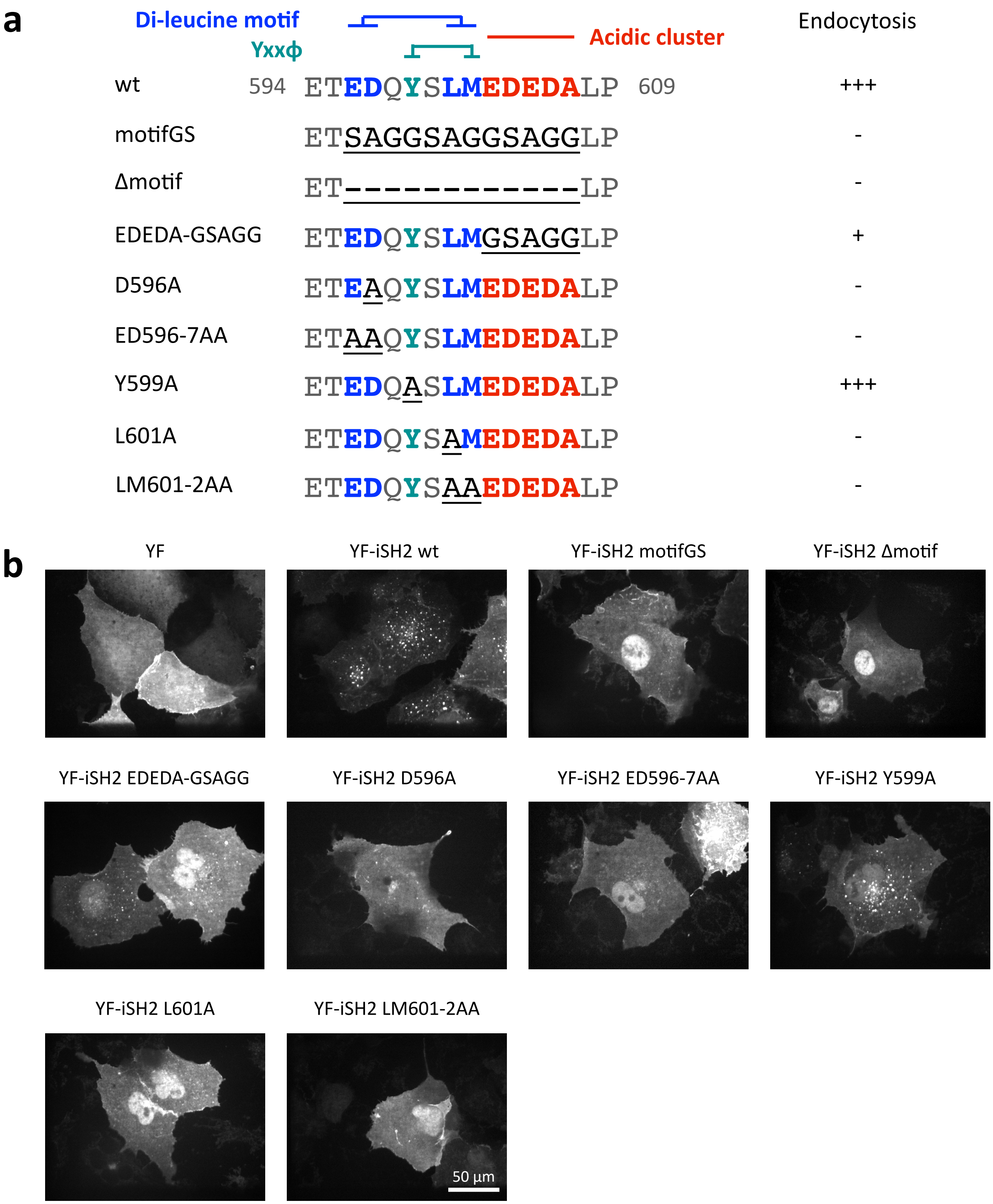

Supplementary Figure 5: Vesicle formation with iSH2 variants. (a) List of the tested iSH2 mutants. Underlines indicate mutation sites. Here, wild type is derived from iSH2 domain of mouse p85β. (b) Confocal images of iSH2-vesicles produced with wild type and mutant iSH2. Cos7 cells were transiently transfected with Lyn-ECFP-FRB, EYFP-FKBP-iSH2, and mCherry-PH(Akt). 15 min after adding 100 nM rapamycin, cells were chilled, washed, and fixed with 4% paraformaldehyde and 0.15% glutaraldehyde.

Supplementary Figure 5

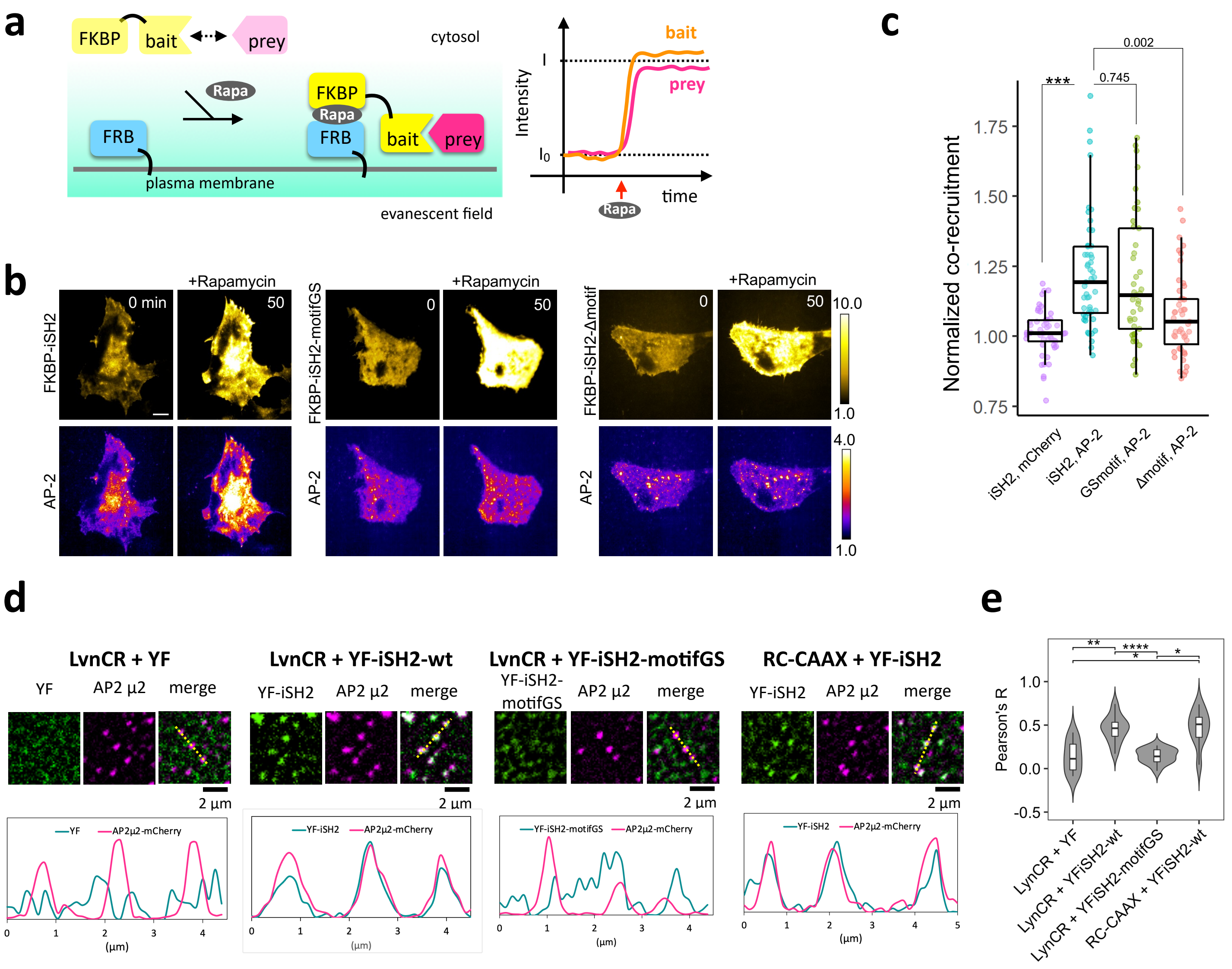

Supplementary Figure 6: iSH2 recruits AP2 to plasma membrane. (a) Schematic of co-recruitment assay. Interaction between bait and prey was evaluated by rapamycin-dependent increase in plasma membrane intensity of prey, here AP2-mCherry. (b) Representative images showing changes in TIRF fluorescence intensities on plasma-membrane recruitment of EYFP-FKBP-iSH2, EYFP-FKBP-iSH2-motifGS and EYFP-FKBP-iSH2-Δmotif and corresponding changes in AP2-mCherry intensities. Scale bar: 10 µm. (c) Co-recruitment indices (I/I_0) of mCherry with EYFP-FKBP-iSH2 and of AP2-mCherry with EYFP-FKBP-iSH2, EYFP-FKBP-iSH2-GSmotif and EYFP-FKBP-iSH2-Δmotif using the live cell co-recruitment assay. ***, $P < 0.001$ or as shown, Student's t test. (d) TIRF images showing co-localization between YF-iSH2 and mCherry-AP2(µ2). During live cell imaging, images were taken 1 min after 100 nM rapamycin addition. To reduce noise, median filtered images were subtracted from raw images. Graphs show line scan of dashed lines in merge images. (e) Pearson's correlation between YFP signal and mCherry-AP2(µ2) signal of (d). Calculation was performed on raw images. For each cell, 10 µm (80 pixels) × 10 µm (80 pixels) areas were selected for the quantification. (c, e) Steel-Dwass test. P-values: *: < 0.05, **: < 0.01, ***: < 0.001, ****: < 0.0001.

Supplementary Figure 6

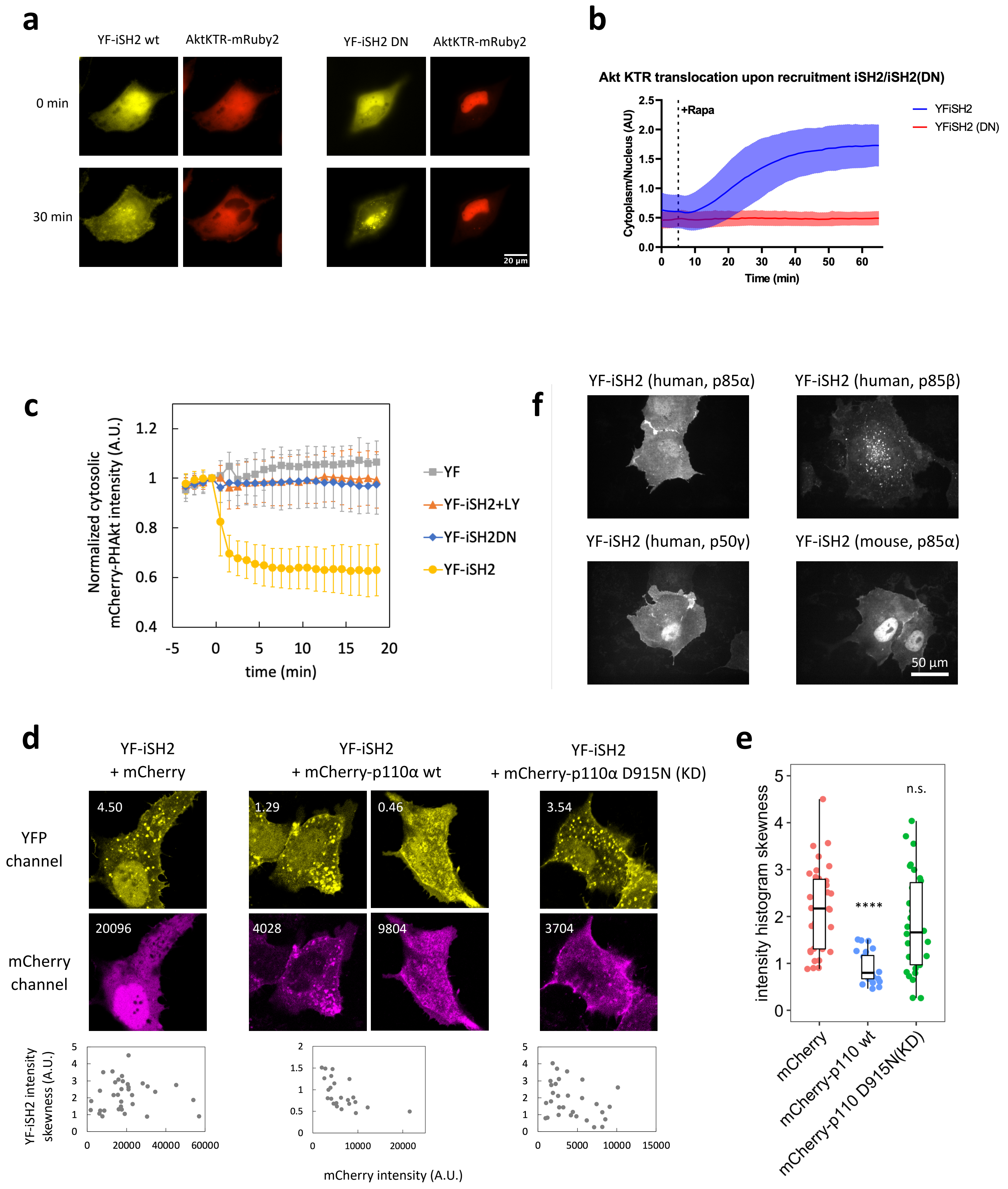

Supplementary Figure 7

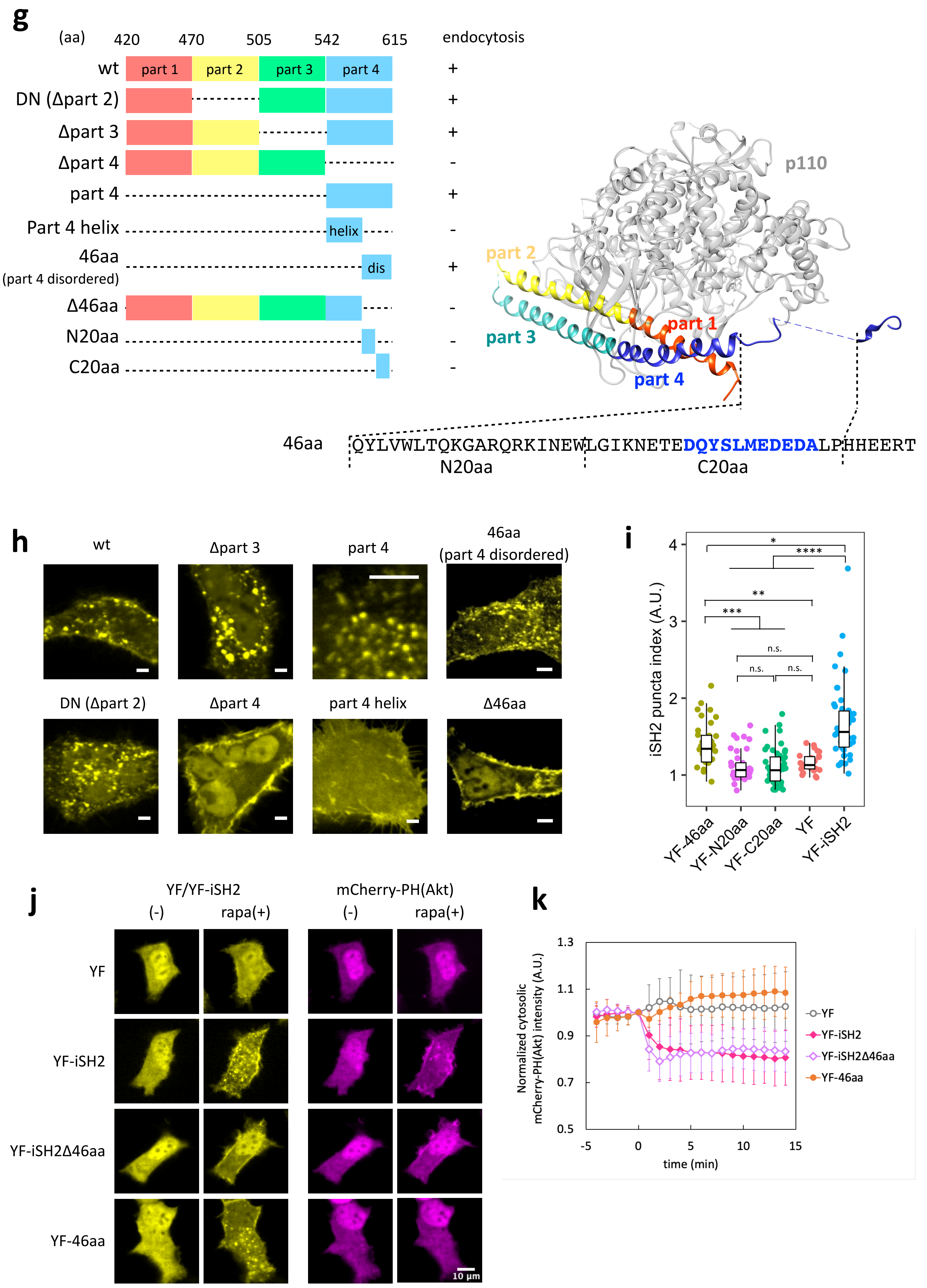
g
(aa)
420
470
505
542
615
endocytosis
wt
part 1
part 2
part 3
part 4
+
DN (Δpart 2)
+
Δpart 3
+
Δpart 4
-
part 4
+
Part 4 helix
helix
-
46aa
(part 4 disordered)
dis
+
Δ46aa
-
N20aa
-
C20aa
-
p110
part 2
part 1
part 3
part 4
46aa
QYLVWLTQKGARQRKINEWLGIKNETEDQYSLMEDEDALPHHEERT
N20aa
C20aa
h
wt
Δpart 3
part 4
46aa
(part 4 disordered)
DN (Δpart 2)
Δpart 4
part 4 helix
Δ46aa
i
iSH2 puncta index (A.U.)
*
****
**
***
n.s.
n.s.
n.s.
YF-46aa
YF-N20aa
YF-C20aa
YF
YF-iSH2
j
YF/YF-iSH2
mCherry-PH(Akt)
(-)
rapa(+)
(-)
rapa(+)
YF
YF-iSH2
YF-iSH2Δ46aa
YF-46aa
10 μm
k
Normalized cytosolic mCherry-PH(Akt) intensity (A.U.)
time (min)
YF
YF-iSH2
YF-iSH2Δ46aa
YF-46aa

Supplementary Figure 7: iSH2-mediated endocytosis is independent of PI3K catalytic activity and C-terminal 46 aa region is necessary and sufficient. (a) Epi-fluorescence microscopy images of Akt-KTR translocation stimulated by iSH2 plasma membrane targeting. HeLa cells were transfected with Lyn-CR, YF-iSH2 (wt or DN), and Akt-KTR-mRuby2. Time indicates time elapsed after 100 nM rapamycin addition. (b) Cytoplasm/Nucleus ratio of Akt-KTR-mRuby2 intensity. (c) Time course of PH(Akt) translocation of Fig. 2a. Cytosolic intensity of mCherry-PH(Akt) was quantified and normalized by time=0. Error bars represent standard deviation. YF-iSH2, n=30 cells. YF-iSH2 + LY, n=28 cells. YF-iSH2DN, n=27 cells. YF, n=28 cells. (d) Confocal images of iSH2 vesicles in the presence of p110 α expression. Values in YFP images and mCherry images indicate skewness of YF-iSH2 intensity histogram and mCherry intensity, respectively. Values in individual cells were plotted at the bottom. (e) The extent of iSH2 vesicle formation in (d) was quantified by skewness of YFP intensity histogram. p-values against mCherry control were shown. (f) Confocal images of vesicles induced by iSH2 derived from different p85 isoforms. Cos7 cells were transiently transfected with Lyn-ECFP-FRB, EYFP-FKBP-iSH2, and mCherry-PH(Akt). 15 min after adding 100 nM rapamycin, cells were chilled, washed, and fixed with 4% paraformaldehyde and 0.15% glutaraldehyde. (g) Schematic representation of iSH2 truncates. Crystal structure of p110β-iSH2β is derived from PDB 2y3a. (h) Representative confocal image of live-cell plasma membrane recruitment of iSH2 truncates in HeLa expressing Lyn-ECFP-FRB, EYFP-FKBP-iSH2 (truncates), and mCherry-PH(Akt). Scale bar, 5 µm. (i) Quantified iSH2 puncta index of iSH2 truncates tested in Cos7 cells expressing Lyn-ECFP-FRB, EYFP-FKBP-iSH2 (truncates), and mCherry-PH(Akt). YF-46aa, n=38 cells. YF-N20aa, n=39 cells. YF-C20aa, n=48 cells. YF, n=27 cells. YF-iSH2, n=46 cells. Box whisker plots represent median, 1st, 3rd quartiles and 1.5×inter-quartile range. (j) Confocal live-cell images of iSH2-vesicles and PH(Akt) translocation. (k) Time course of PH(Akt) translocation of (j). Cytosolic intensity of mCherry-PH(Aki) was quantified and normalized by time=0. Error bars represent standard deviation. YF, n=17 cells. YF-iSH2, n=41 cells. YF-iSH2Δ46aa, n=39 cells. YF-46aa, n=22 cells. (j, k) Data correspond with Fig. 2c. (e, i) p-values (Steel-Dwass test): *: < 0.05, **: < 0.01, ***: < 0.001, ****: < 0.0001. n.s.: not significant.

Supplementary Figure 7

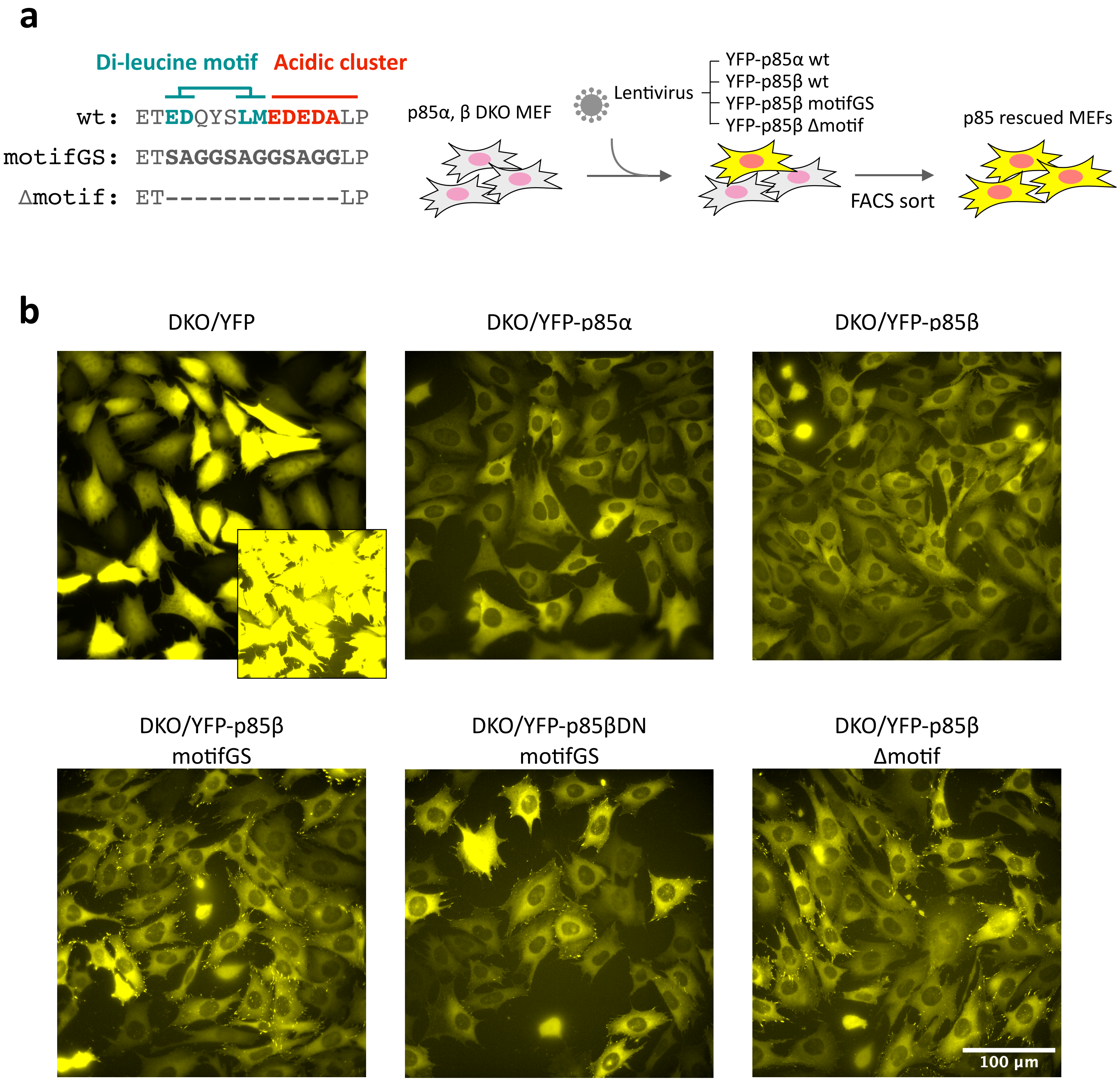

Supplementary Figure 8

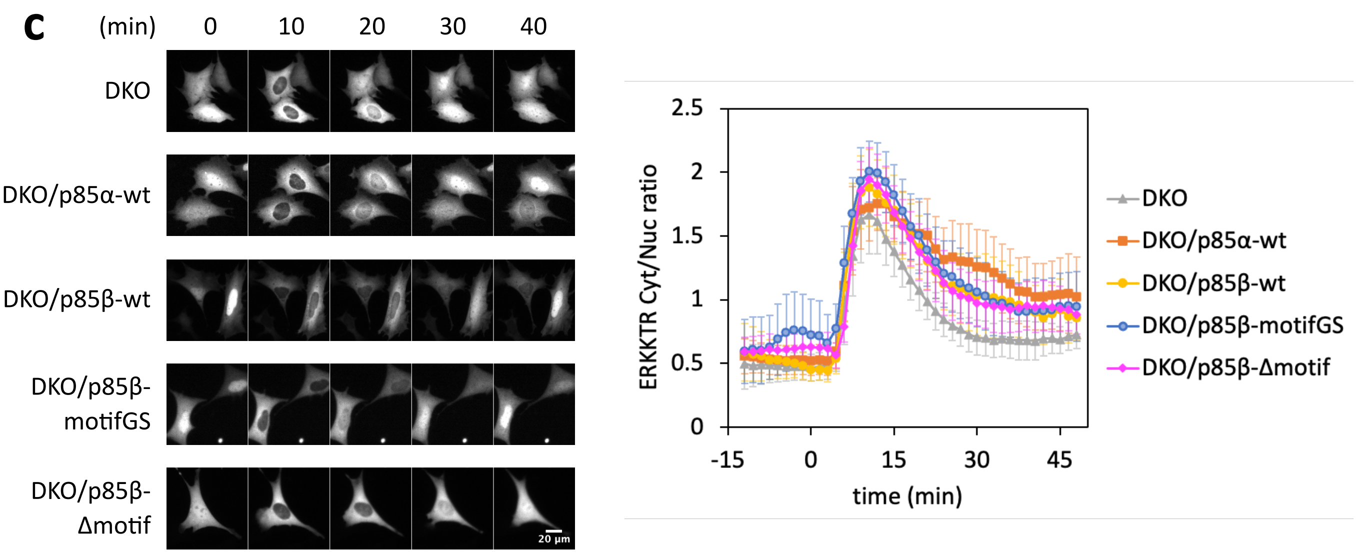

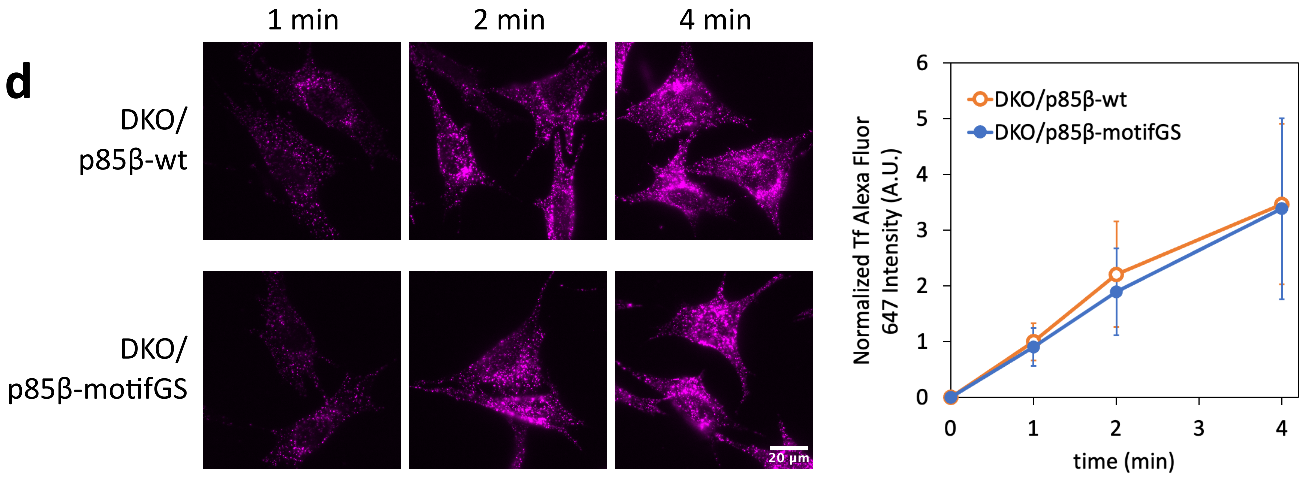

Supplementary Figure 8: Generation and Functional analysis of p85-rescued MEFs. (a) p85α, β double knockout (DKO) MEFs were infected with lentiviruses encoding YFP-p85 variants. Infected cells were FACS-sorted by YFP fluorescence. (b) Epi-fluorescence microscopy images of each cell lines. The same dynamic range was applied to DKO/YFP inset and other cell lines. c) ERK response to PDGF stimulation. Each cell lines was transiently transfected with mCherry-ERKKTR. The cells were serum starved and stimulated with 50 ng/mL PDGF-BB. ERKKTR response was recorded by live cell imaging at 37°C with 5% CO_2. Left: epi-fluorescence microscopy images of mCherry-ERKKTR. Right: quantified Cytosol/Nucleus ratio of mCherry-ERKKTR. Error bars represent 2×SEM (95% CI). DKO, n=18 cells. DKO/p85α-wt, n=18 cells. DKO/p85β-wt, n=18 cells. DKO/p85β-motifGS, n=19 cells. DKO/p85β-Δmotif, n=19 cells. (d) Transferrin uptake. Alexa Fluor 647-conjugated transferrin was added to serum starved cells. After the indicated time, the cells were chilled, washed with acid, and fixed with 4% paraformaldehyde. Left: epi-fluorescence microscopy images of Alexa Fluor 647-conjugated transferrin. Right: quantified Alexa Fluor 647 intensity. Error bars represent standard deviation. n>61 cells for each time point.

Supplementary Figure 8

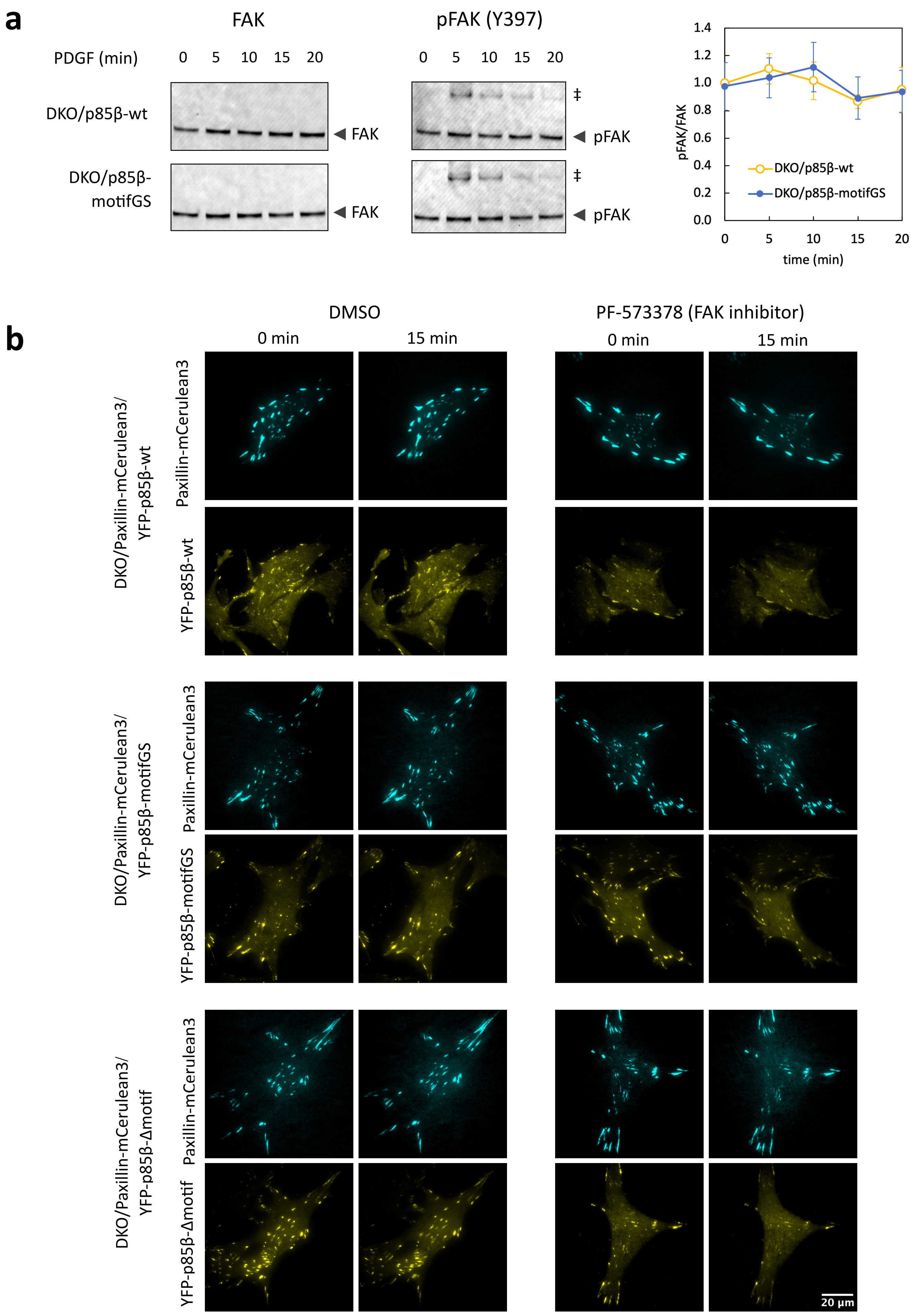

Supplementary Figure 9

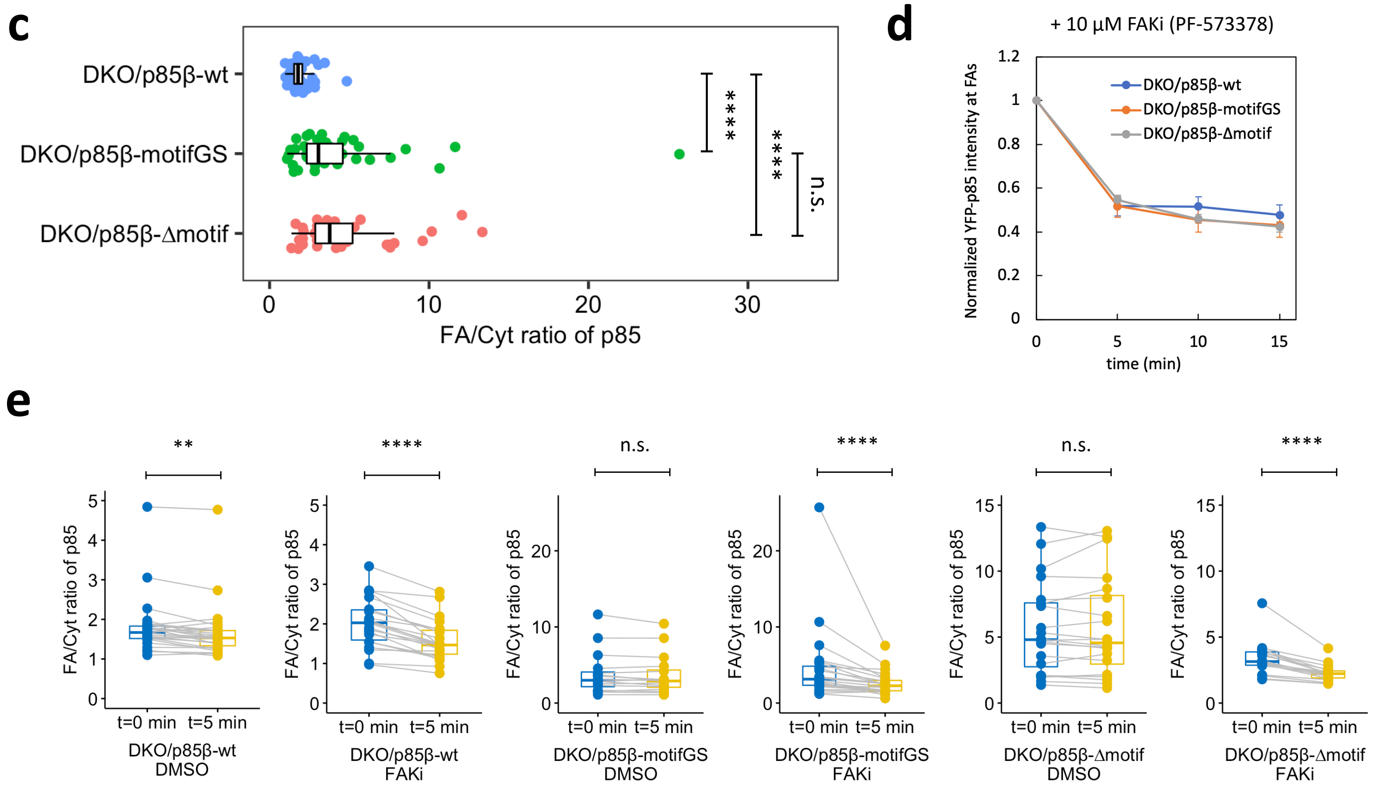

Supplementary Figure 9: PF-573378 (FAK inhibitor) response of p85 variants. (a) Western blot of total- and phospho-FAK (Y397) and its quantification. Cells were treated with 50 ng/mL PDGF for indicated time. pFAK/FAK level was normalized to DKO/p85β-wt 0 min. Error bars represent SEM. ‡ indicates a non-specific band. (b) TIRF images of MEFs stably expressing Paxillin-mCerulean3 and YFP-p85 variants. After serum starvation for one hour, the cells were imaged at 37°C with 5% CO_2 in the imaging media. At time 0, either 10 µM PF-573378 or DMSO was added. (c) Focal adhesion to cytosolic intensity ratio of YFP-p85 measured from the images in (b) under serum starved condition (time=0). (d) Time course of normalized YFP-p85 intensity at focal adhesions after 10 µM PF-573378 addition. YFP-p85 intensity at focal adhesion was measured with image masks created by Paxillin-mCerulean3 images and normalized by time=0. Error bars represent standard deviation. DKO/p85β-wt, n=20 cells. DKO/p85β-motifGS, n=22 cells. DKO/p85β-Δmotif, n=18 cells. (e) Focal adhesion to cytosolic intensity ratio of YFP-p85 in each cell line before and after DMSO or 10 µM PF-573378 (FAK inhibitor) treatment. (c) Steel-Dwass test, (e) paired samples Wilcoxon rank sum test. P-values: *: < 0.05, **: < 0.01, ***: < 0.001, ****: < 0.0001. n.s.: not significant.

Supplementary Figure 9

a

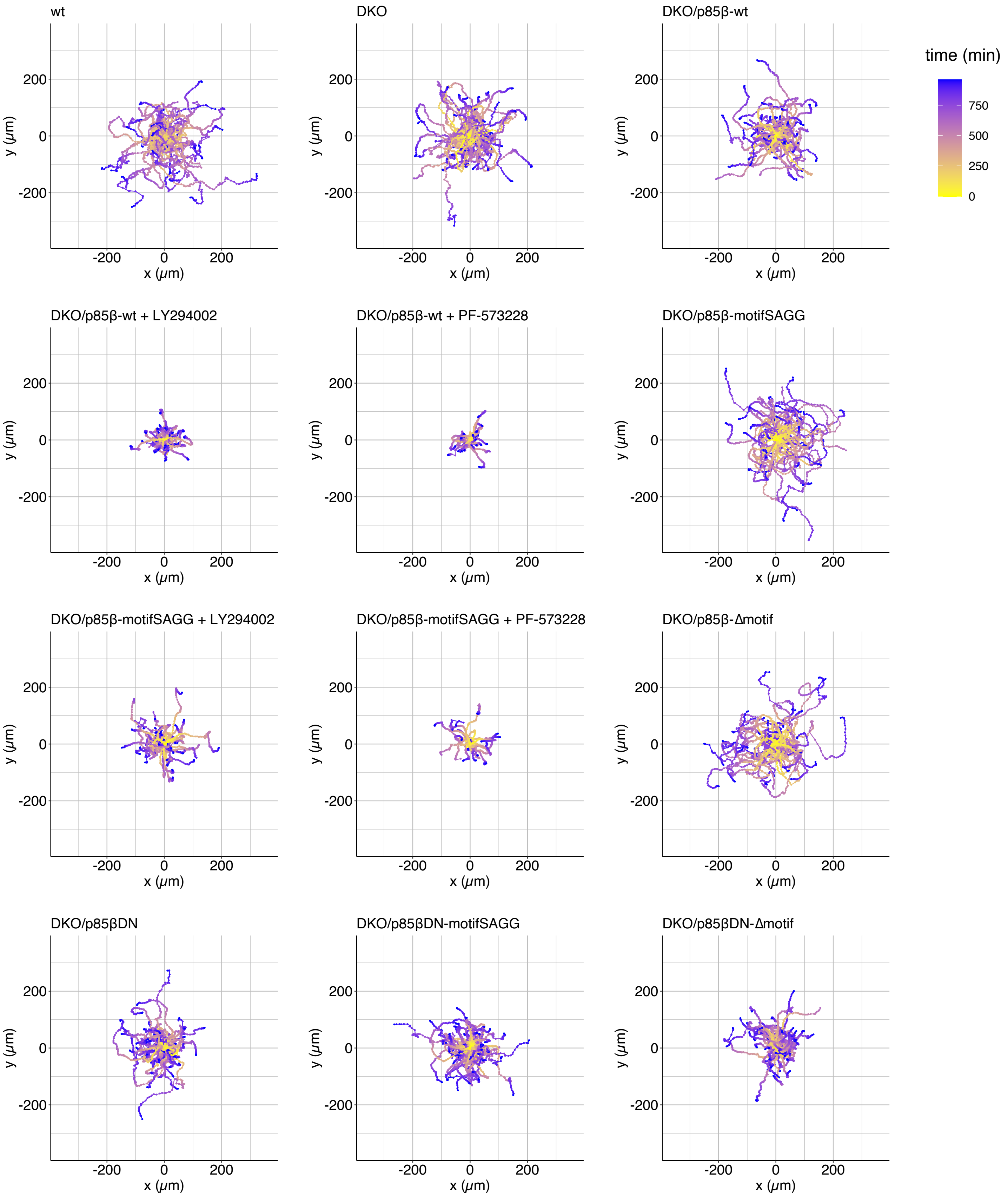

Supplementary Figure 10

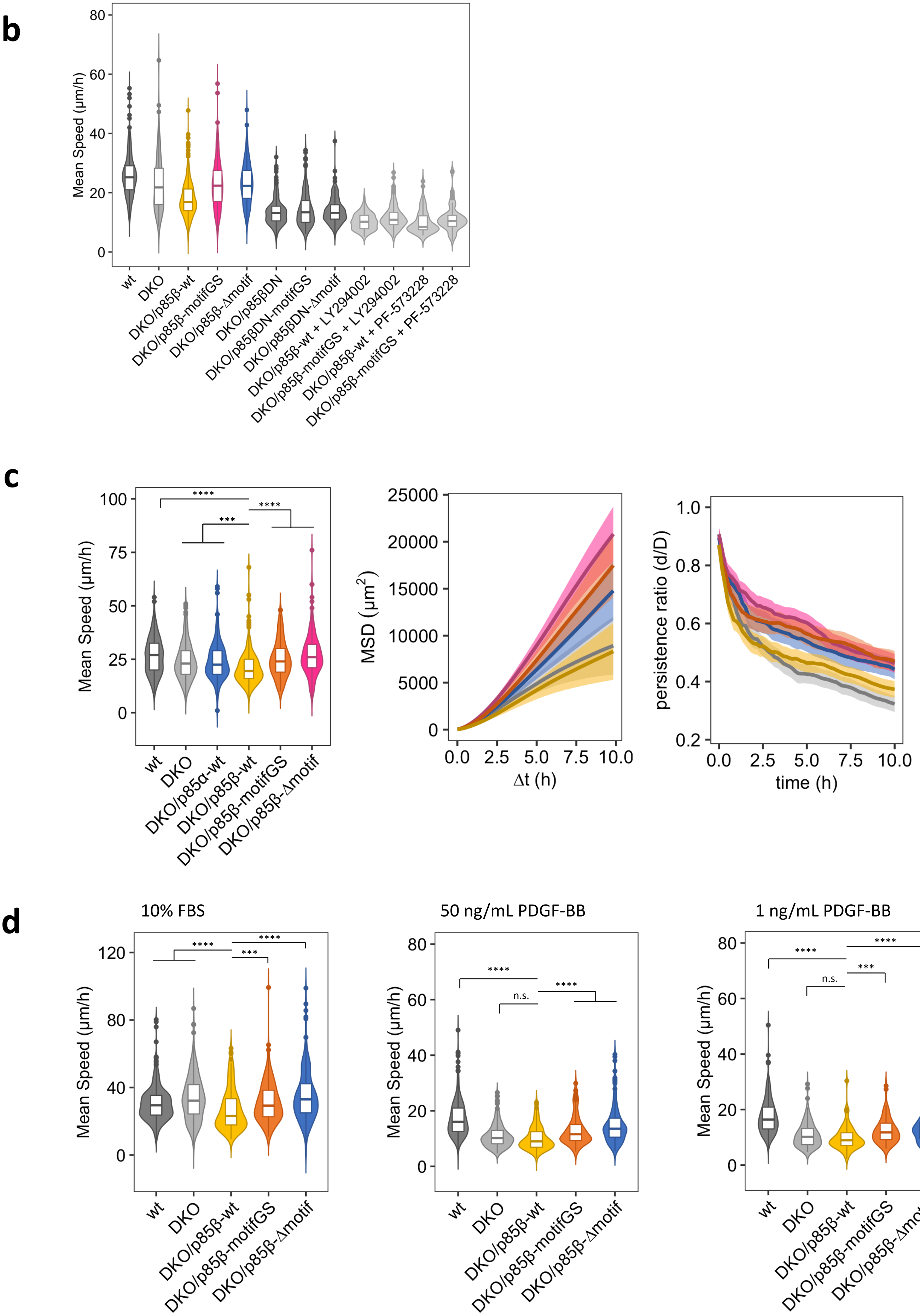

Supplementary Figure 10

e

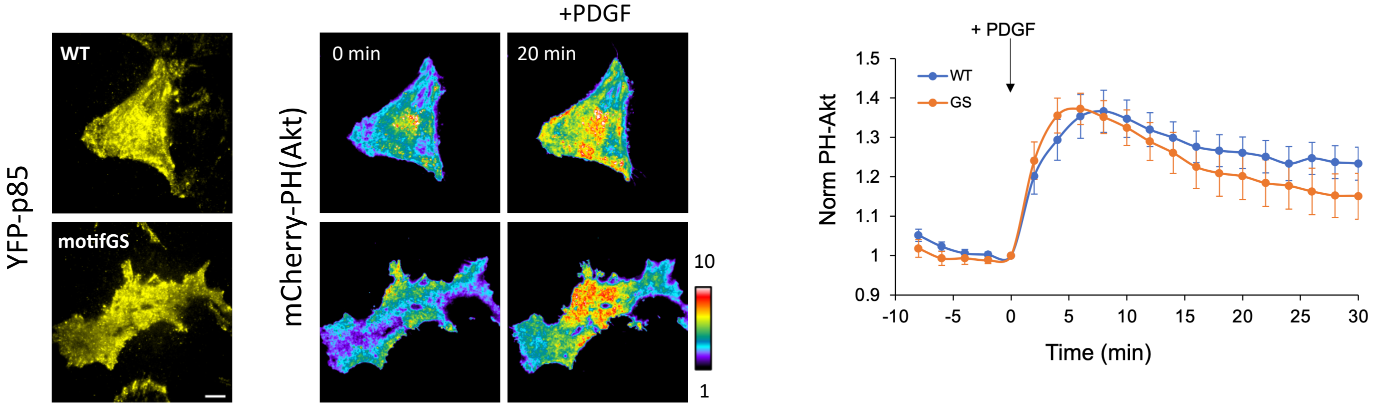

Supplementary Figure 10: Supplementary data of migration assay. (a) Representative tracks of 2D random migration on fibronectin coated plates. (b) Mean speed of each condition of (a). PI3K inhibitor LY294002 and FAK inhibitor PF-573228 were added at 50 µM and 10 µM, respectively. Data set of (a, b) correspond with Fig. 4b-d. (c) Different data set of random migration including DKO/p85α-wt. MSD: mean square displacement. (d) Different data set of random migration stimulated by 10%FBS and 50 or 1 ng/mL PDGF. (e) p85 KO MEF cells rescued with YFP-p85 or YFP-p85-GS-motif and stably expressing mCherry-PH-Akt were treated with PDGF-BB (50 ng/ml final conc.) and imaged in a TIRF microscope to measure changes in mCherry-PH-Akt signal. PDGF-BB was added at time 0. Representative images displaying EYFP-p85 and changes in PH-Akt-mCherry signal in the TIRF plane are shown, scale bar: 10 µm. WT: 77, GS: 52 cells, mean ± 95% C.I. are shown. (c, d) Steel-Dwass test was performed and p-values against DKO/p85β-wt are indicated. P-values: ****: < 0.0001. ***: < 0.001. n.s.: not significant.

Supplementary Figure 10
