# Supplementary material for: Non-catalytic role of phosphoinositide 3-kinase in mesenchymal cell migration through non-canonical induction of p85β/AP2-mediated endocytosis
Source: Nat Commun. 2024 Mar 23;15:2612. doi: 10.1038/s41467-024-46855-y (PMC10960865; doi:10.1038/s41467-024-46855-y)
Supplement: Supplementary file 3 — Description of Additional Supplementary Files [file 41467_2024_46855_MOESM3_ESM.pdf]

Description of Additional Supplementary Files

File Name: Supplementary Data 1
Description: List of plasma membrane anchors used in Supplementary Figure 4d, e.

File Name: Supplementary Data 2
Description: Sequence information of Lyn-ECFP-FRB, FRB-ECFP-KRas4BCAAX, EYFP-FKBP, EYFP-FKBP-iSH2(mouse, beta), and mCherry-PH(Akt).

File Name: Supplementary Movie 1
Description: Confocal images of endocytic vesicles produced by plasma membrane targeting of iSH2 domain. HeLa cells were transiently transfected with Lyn-ECFPFRB, EYFP-FKBP-iSH2, and mCherry-PH(Akt). Imaging was performed at 37°C with 5% CO2. 100 nM rapamycin was added at indicated time.

File Name: Supplementary Movie 2
Description: Confocal images of EYFP-FKBP negative control. HeLa cells were transiently transfected with Lyn-ECFP-FRB, EYFP-FKBP, and mCherry-PH(Akt). Imaging was performed at 37°C with 5% CO2. 100 nM rapamycin was added at indicated time.

File Name: Supplementary Movie 3
Description: Confocal images of room temperature control. HeLa cells were transiently transfected with Lyn-ECFP-FRB, EYFP-FKBP-iSH2, and mCherry-PH(Akt). Imaging was performed at 23°C with 5%CO2. 100 nM rapamycin was added at indicated time.
