# Supplementary material for: Non-catalytic role of phosphoinositide 3-kinase in mesenchymal cell migration through non-canonical induction of p85β/AP2-mediated endocytosis
Source: Nat Commun. 2024 Mar 23;15:2612. doi: 10.1038/s41467-024-46855-y (PMC10960865; doi:10.1038/s41467-024-46855-y)
Supplement: Supplementary file 9 — Reporting Summary [file 41467_2024_46855_MOESM9_ESM.pdf]

nature portfolio

Corresponding author(s): Hideaki Matsubayashi, Takanari Inoue

Last updated by author(s): Mar 1, 2024

Reporting Summary

Nature Portfolio wishes to improve the reproducibility of the work that we publish. This form provides structure for consistency and transparency in reporting. For further information on Nature Portfolio policies, see our Editorial Policies and the Editorial Policy Checklist.

Statistics

For all statistical analyses, confirm that the following items are present in the figure legend, table legend, main text, or Methods section.

n/a	Confirmed	
☐	☒	The exact sample size (*n*) for each experimental group/condition, given as a discrete number and unit of measurement
☐	☒	A statement on whether measurements were taken from distinct samples or whether the same sample was measured repeatedly
☐	☒	The statistical test(s) used AND whether they are one- or two-sided<br>*Only common tests should be described solely by name; describe more complex techniques in the Methods section.*
☒	☐	A description of all covariates tested
☐	☒	A description of any assumptions or corrections, such as tests of normality and adjustment for multiple comparisons
☐	☒	A full description of the statistical parameters including central tendency (e.g. means) or other basic estimates (e.g. regression coefficient) AND variation (e.g. standard deviation) or associated estimates of uncertainty (e.g. confidence intervals)
☐	☒	For null hypothesis testing, the test statistic (e.g. *F*, *t*, *r*) with confidence intervals, effect sizes, degrees of freedom and *P* value noted<br>*Give P values as exact values whenever suitable.*
☒	☐	For Bayesian analysis, information on the choice of priors and Markov chain Monte Carlo settings
☒	☐	For hierarchical and complex designs, identification of the appropriate level for tests and full reporting of outcomes
☐	☒	Estimates of effect sizes (e.g. Cohen's *d*, Pearson's *r*), indicating how they were calculated

Our web collection on statistics for biologists contains articles on many of the points above.

Software and code

Policy information about availability of computer code

Data collection	Metamorph 7.10.2 (Molecular Devices), NIS - Elements AR 5.02.01, ZEN (Zeiss), LocalColabFold ver. 1.5.2, NAMD ver. 3.0b4105.
Data analysis	Fiji version 2.3.0/1.53q, R version 4.1.1, Visual Molecular Dynamics (VMD) software.

For manuscripts utilizing custom algorithms or software that are central to the research but not yet described in published literature, software must be made available to editors and reviewers. We strongly encourage code deposition in a community repository (e.g. GitHub). See the Nature Portfolio guidelines for submitting code & software for further information.

Data

Policy information about availability of data

All manuscripts must include a data availability statement. This statement should provide the following information, where applicable:

- Accession codes, unique identifiers, or web links for publicly available datasets
- A description of any restrictions on data availability
- For clinical datasets or third party data, please ensure that the statement adheres to our policy

The datasets generated during this study are available from the corresponding author upon request. Source data are provided with this paper. Structure of PI3K and AP2 complex were obtained from the previously published PDB data, 2Y3A [https://doi.org/10.2210/pdb2Y3A/pdb], 2XA7 [https://doi.org/10.2210/pdb2XA7/pdb], and 2JKR [https://doi.org/10.2210/pdb2JKR/pdb].

Research involving human participants, their data, or biological material

Policy information about studies with human participants or human data. See also policy information about sex, gender (identity/presentation), and sexual orientation and race, ethnicity and racism.

Reporting on sex and gender	N/A
Reporting on race, ethnicity, or other socially relevant groupings	N/A
Population characteristics	N/A
Recruitment	N/A
Ethics oversight	N/A

Note that full information on the approval of the study protocol must also be provided in the manuscript.

Field-specific reporting

Please select the one below that is the best fit for your research. If you are not sure, read the appropriate sections before making your selection.

☒ Life sciences ☐ Behavioural & social sciences ☐ Ecological, evolutionary & environmental sciences

For a reference copy of the document with all sections, see nature.com/documents/nr-reporting-summary-flat.pdf

Life sciences study design

All studies must disclose on these points even when the disclosure is negative.

Sample size	No sample size estimate was performed, and our sample sizes are consistent with that typically used in live-cell imaging experiments and other biology experiments. References: doi: 10.1038/s41592-020-0913-x, doi: 10.1038/s41589-021-00791-w
Data exclusions	For cell migration analysis, we excluded dead cells based on morphology, motionless behavior, and aberant Hoechist intensity. For PH(Akt) translocation assay, we only analyzed cells with clear YF-iSH2 translocation to evaluate the effect of YF-iSH2. In co-recruitment assay (Extended Figure 6), we excluded two outliers which had significant morphological changes.
Replication	At least three replicates were performed for all quantified experiments except for Supplementary Figure 10d. All the attempts at the replication were successful.
Randomization	No randomization was used because the study does not involve allocation into different experimental groups.
Blinding	No blinding was used. Blinding was not relevant to the study because samples were not grouped and randomized.

Reporting for specific materials, systems and methods

We require information from authors about some types of materials, experimental systems and methods used in many studies. Here, indicate whether each material, system or method listed is relevant to your study. If you are not sure if a list item applies to your research, read the appropriate section before selecting a response.

Materials & experimental systems

n/a	Involved in the study	
☐	☒	Antibodies
☐	☒	Eukaryotic cell lines
☒	☐	Palaeontology and archaeology
☒	☐	Animals and other organisms
☒	☐	Clinical data
☒	☐	Dual use research of concern
☒	☐	Plants

Methods

n/a	Involved in the study	
☒	☐	ChIP-seq
☒	☐	Flow cytometry
☒	☐	MRI-based neuroimaging

Antibodies

Antibodies used	Anti-Vinculin (Sigma-Aldrich, MAB3574-25UG), anti-Akt (Cell signaling, 9272S), anti-phospho-Akt (T308) (Cell signaling, 13038S), anti-FAK (Cell signaling, 13009S), anti-phospho-FAK (Y397) (Cell signaling, 8556S), anti-GAPDH antibody (Santa Cruz, sc-32233), Alexa Fluor 488-conjugated anti-Rabbit IgG (Thermo Fisher Scientific, A-21206), Alexa Fluor 568-conjugated anti-Mouse IgG (Thermo Fisher

	Scientific, A11004), Alexa Fluor 647-conjugated anti-Mouse IgG (Thermo Fisher Scientific, A-31571), anti-Adaptin α (BD Biosciences, BD610501), Anti-IgG (H+L chain) (Mouse) pAb-HRP (MBL 330).
Validation	Anti-Akt (Cell signaling, 9272S): Specific detection was confirmed by western blot and immunofluorescence of mammalian cells by manufacturer. Species reactivity information of the manufacture includes mouse. Anti-phospho-Akt (T308) (Cell signaling, 13038S): Specific detection was confirmed by western blot and immunofluorescence of mammalian cells by manufacturer. Species reactivity information of the manufacture includes mouse. anti-FAK (Cell signaling, 13009S): Specific detection was confirmed by western blot of mammalian cells by manufacturer. Species reactivity information of the manufacture includes mouse. anti-phospho-FAK (Y397) (Cell signaling, 8556S): Specific detection was confirmed by western blot of mammalian cells by manufacturer. Reactivity was confirmed by Supplementary data in this manuscript. Anti-Adaptin alpha (BD Biosciences, BD610501): Specific detection was confirmed by western blot and immunofluorescence of mammalian cells by manufacturer. Species reactivity information of the manufacture includes mouse.

Eukaryotic cell lines

Policy information about cell lines and Sex and Gender in Research

Cell line source(s)	HeLa, COS-7 cells were derived from ATCC. HEK293FT was a kind gift from Andrew Ewald lab. WT and p85 double knockout MEF cells were kind gift from Brendan Manning lab.
Authentication	No further authentication was done.
Mycoplasma contamination	Mycoplasma testing was not done in all cell lines used in this study.
Commonly misidentified lines (See ICLAC register)	No commonly misidentified lines.

Plants

Seed stocks	N/A
Novel plant genotypes	N/A
Authentication	N/A
